# Machine learning models predict coagulopathy in traumatic brain injury patients in ER

**DOI:** 10.3389/fneur.2025.1649869

**Published:** 2025-09-18

**Authors:** Haoyu Wang, Wenying Cao, Jianhuang Huang, Yuxing Feng, Cheng Li

**Affiliations:** ^1^Department of Neurosurgery, Chongqing Ninth People's Hospital, Chongqing, China; ^2^Department of Neurology, The Ninth People’s Hospital of Chongqing, Chongqing, China; ^3^Department of Neurosurgery, Affiliated Hospital of Putian University, Fujian, China

**Keywords:** traumatic brain injury, coagulopathy, machine learning, emergency medicine, feature importance, SHAP analysis, risk stratification

## Abstract

Traumatic brain injury (TBI) is a critical emergency condition, with 15–35% of patients developing coagulopathy, increasing risks of secondary brain injury and mortality. We developed a machine learning model to predict coagulopathy in TBI patients in the emergency room. Using data from 322 TBI patients (mean age 55.7 ± 21.1 years, coagulopathy incidence 15.8%) at Chongqing Ninth People’s Hospital (2018–2024), we collected clinical and laboratory data (GCS scores, blood counts, liver function). Data were preprocessed in R, using SMOTE for class imbalance and selecting top 70% features by information gain. Among 11 algorithms, Random Forest (RF) achieved the best performance (AUC = 0.92, recall = 0.94, false negative rate = 6%), outperforming coagulation tests. Neutrophil percentage, A/G ratio, and ALT were key predictors, reflecting inflammation and liver dysfunction. SHAP analysis enhanced model interpretability. This model supports rapid risk stratification for early intervention, though multi-center validation is needed.

## 1 Introduction

Traumatic brain injury (TBI) is one of the most common critical conditions in emergency departments. Globally, an estimated 50 million TBI cases occur annually, significantly increasing mortality and disability risks and presenting a major public health challenge (1–3). Research indicates that approximately 35% of TBI patients exhibit coagulopathy upon admission (4–6), which can lead to secondary brain injuries (such as hematoma expansion and new hemorrhages), substantially worsening patient outcomes (5, 7, 8). Therefore, timely identification and management of coagulopathy is not only a core component of emergency TBI treatment but directly impacts patient prognosis (9–11).

Currently, coagulation function assessment relies on traditional testing methods such as prothrombin time (PT) and activated partial thromboplastin time (APTT). However, these methods are time-consuming (typically requiring tens of minutes to hours) and have limited sensitivity for trauma-induced coagulopathy (12–15). Recent studies have revealed that TBI-related coagulopathy is closely associated with multiple factors including inflammatory response, vascular endothelial damage, and impaired hepatic synthesis of coagulation factors (5, 16, 17). These complex mechanisms necessitate rapid, comprehensive predictive tools to meet the time-sensitive demands of emergency care.

Machine learning (ML) can integrate high-dimensional data to discover predictive patterns that traditional statistical methods struggle to capture, demonstrating superior potential to conventional models in trauma medicine. While recent ML applications in emergency risk stratification have significantly improved prediction accuracy, research on rapid prediction of TBI-associated coagulopathy remains limited, with existing models often lacking interpretability or real-time applicability. This study aims to develop and validate a machine learning-based prediction model using routine clinical and laboratory data available in emergency departments to rapidly identify high-risk TBI patients for coagulopathy, supporting early intervention and facilitating precise emergency management.

## 2 Methods

### 2.1 Data source

**Figure 1:**
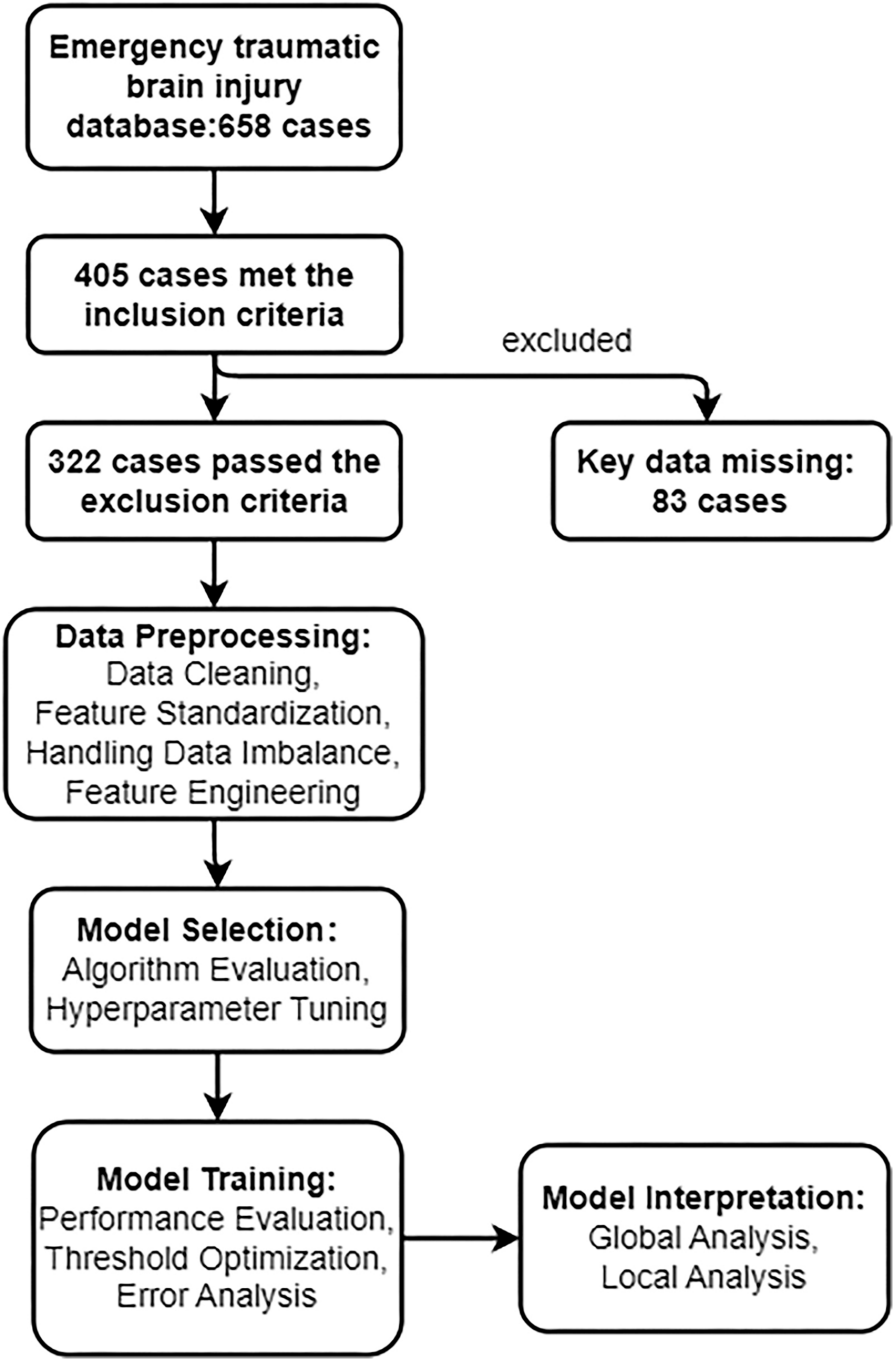
Technical roadmap of this study.

This study utilized the TBI database from Chongqing Ninth People’s Hospital, collecting clinical and laboratory data from emergency department TBI patients between January 2018 and December 2024. The target was to predict whether patients would develop coagulopathy (binary variable). Coagulopathy was defined as elevated International Normalized Ratio (INR ≥ 1.2) or prolonged activated partial thromboplastin time (APTT, reference range 28–34 s) (14, 18, 19). Clinical features collected included demographic information (e.g., age, gender), comorbidities (e.g., hypertension, diabetes), trauma-related indicators (e.g., GCS score, TBI type, injury location), and laboratory results (e.g., complete blood count, liver and kidney function tests, electrolytes). Inclusion criteria were: age≥18 years, CT-confirmed TBI, and complete laboratory data within 2 h of admission. Exclusion criteria included: severe non-cranial polytrauma, pre-existing coagulation disorders (e.g., hemophilia), use of anticoagulant medications (to avoid confounding factors), or missing critical data. This study was approved by the Ethics Committee of Chongqing Ninth People’s Hospital (Ethics approval number: 2025011). Due to the retrospective design of this study, the Ethics Committee waived the requirements for informed consent and clinical trial registration. All data were collected and analyzed anonymously, with no potential harm to patients. This research was conducted in accordance with the Declaration of Helsinki (2013 revision). Figure 1 illustrates the technical roadmap of this study.

### 2.2 Data preprocessing

Data preprocessing was completed in R 4.3.3 environment. The dataset was split in a 7:3 ratio (stratified sampling to ensure balanced coagulopathy proportions) into training and testing sets. The R package “dplyr” (V1.1.4) (20) was used to handle missing values, with numerical variables imputed using median values and categorical variables imputed using mode values. Categorical variables were converted to numerical form through one-hot encoding. Numerical features were centered (mean = 0) and standardized (standard deviation = 1) using “mlr3pipelines” (V0.7.2) (21). To address data imbalance, the R package “smotefamily” (V1.4.0) (22) applied the “SMOTE” technique (K = 5, replication factor = 5) to generate synthetic samples, and 70% of the most predictive features were selected based on information gain, removing zero-variance features. The final dataset was defined as a binary classification task (ID “Coagulation”) through the R package “mlr3” (V0.23.0) (23) framework, using 5-fold cross-validation to divide training and validation data.

### 2.3 Model selection and rapid screening

To select appropriate machine learning algorithms, the mlr3 framework was used to preliminarily evaluate the default parameter performance of 11 models, including Random Forest (RF), Gradient Boosting Machine (GBM), XGBoost, Elastic Net Regularized Logistic Regression, Naive Bayes, Decision Tree, Logistic Regression, k-Nearest Neighbors (k-NN), Support Vector Machine (SVM), Linear Discriminant Analysis (LDA), and Single-Layer Neural Network. The evaluation process employed 5-fold cross-validation, with Area Under the Curve (AUC) as the primary metric, supplemented by accuracy, precision, recall, and F1 score. The best-performing Random Forest, Gradient Boosting Machine, and Support Vector Machine were selected for hyperparameter tuning.

### 2.4 Hyperparameter tuning

Random Forest optimization parameters included number of trees (100–500), number of features for splitting (2–12), minimum node size (1–8), and maximum number of nodes (3–10), using random search (50 iterations) via the R package “mlr3tuning” (V1.3.0) (24). Gradient Boosting Machine optimized number of trees (50–1,000), interaction depth (3–10), and learning rate (0.01–0.1) using the Hyperband algorithm (25). Support Vector Machine optimized kernel type (linear/polynomial/radial), regularization parameter (0.1–10), kernel parameter (0.01–1), and polynomial kernel degree (2–5) using random search. Tuning targeted AUC, incorporating 5-fold cross-validation and comprehensively evaluating AUC, accuracy, precision, recall, and F1 score. The best-performing model was selected for further analysis.

### 2.5 Final model training and evaluation

Random Forest, showing the best performance, was selected as the final model, with optimal hyperparameters (number of trees = 225, features for splitting = 8, minimum node size = 2, maximum nodes = 5) used for retraining on the complete dataset, configuring probability output to support subsequent analysis, implemented through the mlr3 framework. Model performance was evaluated through 5-fold cross-validation, reporting mean and median values of AUC, accuracy, precision, recall, and F1 score, along with confusion matrix analysis of classification error distribution. Out-of-bag error of Random Forest was used to verify the adequacy of decision tree quantity. The model output coagulopathy probability, optimized based on the ROC curve, with a probability threshold of 0.6 (corresponding to SHAP value>0.7) selected to balance sensitivity and specificity.

### 2.6 Model interpretation

To enhance model interpretability, global and local analysis methods were employed. At the global level, the DALEX package calculated feature contributions to predictions through Dropout Loss, with results visualized as bar charts using the R package “ggplot2” (V3.5.1) (26). At the local level, the R package “shapviz” (V0.9.7) (27) calculated SHAP values for 150 random samples, analyzing feature contributions to individual predictions, visualized with bee swarm plots and force plots, with the background dataset randomly sampled from 100 preprocessed data points.

### 2.7 Statistical analysis

Statistical description and analysis were completed in R 4.3.3 environment. Continuous variables were described as mean±standard deviation, categorical variables as frequency (percentage), group comparisons used Wilcoxon rank-sum test or chi-square test, with significance level set at *p* < 0.05.

## 3 Results

### 3.1 Clinical characteristics of the cohort

Between 2018 and 2024, emergency department admitted 658 acute TBI patients, of whom 405 met preliminary inclusion criteria. After excluding those with missing key data or other disqualifying conditions (*n* = 83), 322 patients (mean age 55.7 ± 21.1 years, 61% male) were ultimately included for analysis. Fifty-one patients (15.8%) were diagnosed with coagulopathy. Table 1 summarizes baseline characteristics. Significant clinical differences were observed between coagulopathy and non-coagulopathy groups: (1) Trauma severity: coagulopathy group had significantly lower GCS scores than non-coagulopathy group (7.7 ± 2.7 vs. 9.4 ± 2.5, *p* < 0.001). (2) Inflammatory response: coagulopathy group showed significantly elevated neutrophil percentage (85.0% ± 11.0% vs. 70.0% ± 11.0%, *p* < 0.001). (3) Liver function indicators: coagulopathy group had higher ALT levels (28.1 ± 7.1 vs. 22.3 ± 5.1 U/L, *p* < 0.001) and lower A/G ratio (1.31 ± 0.32 vs. 1.72 ± 0.24, *p* < 0.001). (4) Hematological indicators: coagulopathy group had lower hemoglobin (Hb) levels (104.3 ± 11.7 vs. 117.8 ± 9.8 g/L, *p* < 0.001), and significantly elevated white blood cell count (WBC) and neutrophil count (NEUT) (*p* < 0.001). Other parameters such as serum sodium, potassium, calcium, and gender showed no significant differences (*p* < 0.05, Supplementary Table 1).

**Table 1:** Characteristic of TBI patients with coagulopathy and without coagulopathy.

Features	Overall (*n* = 322)	With coagulopathy (*n* = 271)	Without coagulopathy (*n* = 51)	p*
Age	55.70 ± 21.09	54.58 (20.61)	61.71 ± 22.78	0.028
GCS	9.11 ± 2.57	9.38 ± 2.46	7.65 ± 2.67	<0.001
Hb, g/L	115.70 ± 11.25	117.84 ± 9.81	104.30 ± 11.65	<0.001
WBC,10^9^/L	17.21 ± 5.37	16.46 ± 4.92	21.19 ± 5.92	<0.001
NEUT,10^9^/L	12.99 ± 5.82	11.96 ± 5.06	18.46 ± 6.56	<0.001
NEUT percentage	0.73 ± 0.12	0.70 ± 0.11	0.85 ± 0.11	<0.001
LYMPH percentage	12.10 ± 2.13	12.35 ± 2.08	10.78 ± 1.94	<0.001
ALT, U/L	23.22 ± 5.85	22.31 ± 5.11	28.05 ± 7.08	<0.001
AST, U/L	31.57 ± 5.83	31.91 ± 5.91	29.74 ± 5.07	0.014
A/G	1.66 ± 0.29	1.72 ± 0.24	1.31 ± 0.32	<0.001
Cr, μmol/L	67.26 ± 9.70	64.22 ± 8.12	67.83 ± 9.87	0.014

* *p* < 0.05 by Wilcoxon rank-sum test for continuous variables and *χ*^2^ test for categorical variables.

### 3.2 Model selection and initial performance screening

Using the mlr3 framework, we preliminarily evaluated the default parameter performance of 11 machine learning algorithms (Table 2). Although recall might be the primary consideration for model performance in emergency settings, all models maintained high recall levels (0.93–0.97), so an AUC-based optimization strategy better aligned with clinical risk stratification needs. Results showed that GBM algorithm (AUC = 0.89), RF algorithm (AUC = 0.88), and SVM algorithm (AUC = 0.87) performed best and were selected for subsequent hyperparameter tuning.

**Table 2:** Performance of 11 algorithm models with default parameters.

Algorithm	AUC	Accuracy	Precision	Recall	Fβ score
Random Forest	0.88	0.93	0.95	0.96	0.95
XGBoost	0.84	0.90	0.92	0.96	0.94
Elastic Net Logistic Regression	0.85	0.90	0.92	0.97	0.94
Naive Bayes	0.85	0.92	0.95	0.95	0.95
Gradient Boosting Machine	0.89	0.91	0.93	0.96	0.94
Decision Tree	0.83	0.91	0.93	0.96	0.95
Logistic Regression	0.86	0.87	0.92	0.93	0.92
k-Nearest Neighbors	0.85	0.89	0.91	0.96	0.93
Support Vector Machine	0.87	0.90	0.92	0.96	0.94
Linear Discriminant Analysis	0.86	0.93	0.95	0.96	0.95
Neural Network	0.55	0.84	0.87	0.96	0.91

### 3.3 Optimal model establishment and evaluation

**Figure 2:**
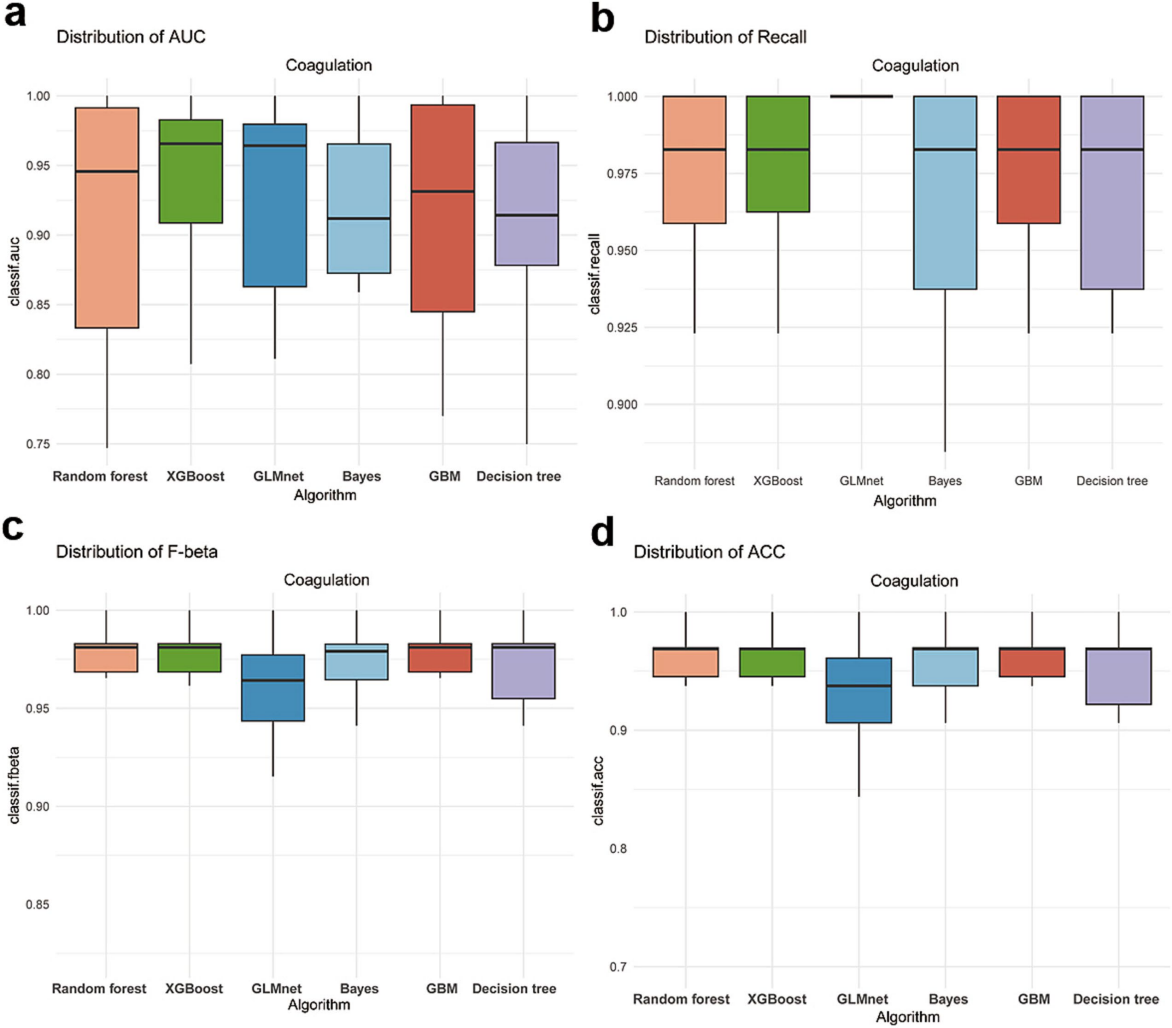
Performance indicators of GBM, SVM and RF algorithms after parameter tuning, with horizontal lines representing median performance indicators under full parameters. **(a)** AUC distribution of various algorithms. **(b)** Recall distribution of various algorithms. **(c)** F-beta distribution of various algorithms. **(d)** ACC distribution of various algorithms.

**Figure 3:**
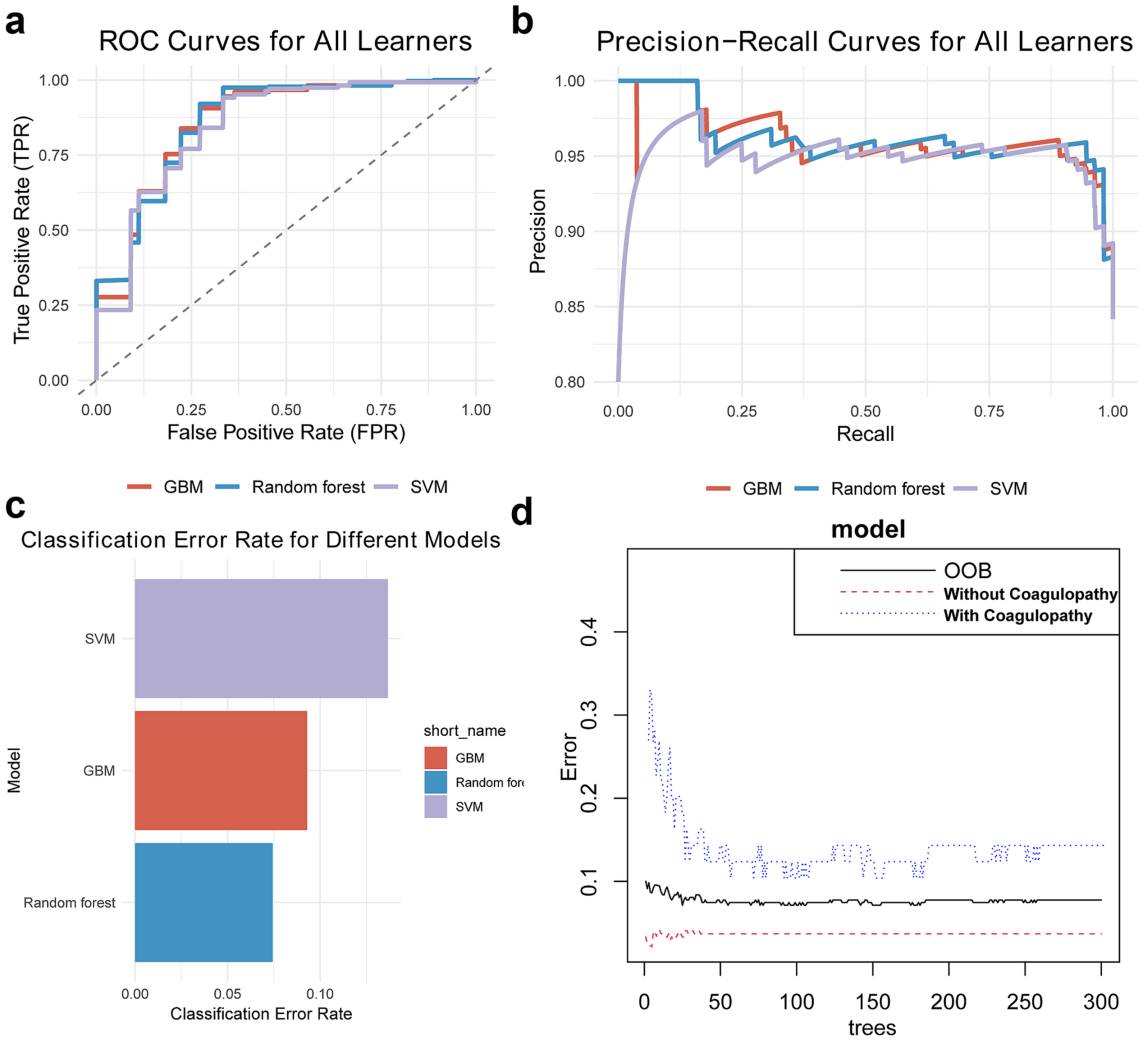
Random Forest model is the optimal model. **(a,b)** AUC and PRC show RF algorithm outperforms GBM and SVM. **(c)** Classification error chart shows RF algorithm has minimal classification error. **(d)** Out-of-bag error chart suggests current RF algorithm parameters are well optimized.

After hyperparameter tuning, we obtained performance parameters for these three algorithms (Table 3). The RF algorithm performed best (Figure 2), with test set AUC improving from 0.87 to 0.92 (ΔAUC = +0.04), and median cross-validation AUC of 0.91 (IQR 0.89–0.93). In comparison, GBM and SVM algorithms improved to AUCs of 0.90 and 0.88 respectively, while RF algorithm training time was only 15% of GBM’s (2.3 min vs. 15 min), making it more suitable for real-time prediction needs in emergency settings. Additionally, AUC and PRC curves showed RF model superiority over GBM and SVM (Figures 3a,b). Classification error analysis indicated (Figure 3c) that RF had a negative prediction error rate as low as 6%, outperforming GBM (9%) and SVM (17%). Therefore, Random Forest algorithm was the optimal model. RF optimal parameters were: number of decision trees = 225, features for splitting = 8, minimum node size = 2. Model robustness analysis showed that when tree number>220, OOB error entered a stable plateau (<0.08), indicating 225 trees sufficiently balanced model complexity and prediction accuracy. Error rates were 0.13 (95%CI 0.10–0.16) for the coagulopathy group and 0.05 (95%CI 0.03–0.07) for the non-coagulopathy group, with McNemar test showing no significant difference between group errors (*p* = 0.12, Figure 3d).

**Table 3:** Performance indicators of optimized random forest, GBM and SVM.

Algorithm	AUC	Accuracy	Precision	Recall	Fβ score
Random Forest	0.92	0.94	0.93	0.94	0.94
Gradient Boosting Machine	0.90	0.92	0.91	0.94	0.93
Support Vector Machine	0.88	0.90	0.90	0.93	0.92

**Figure 4:**
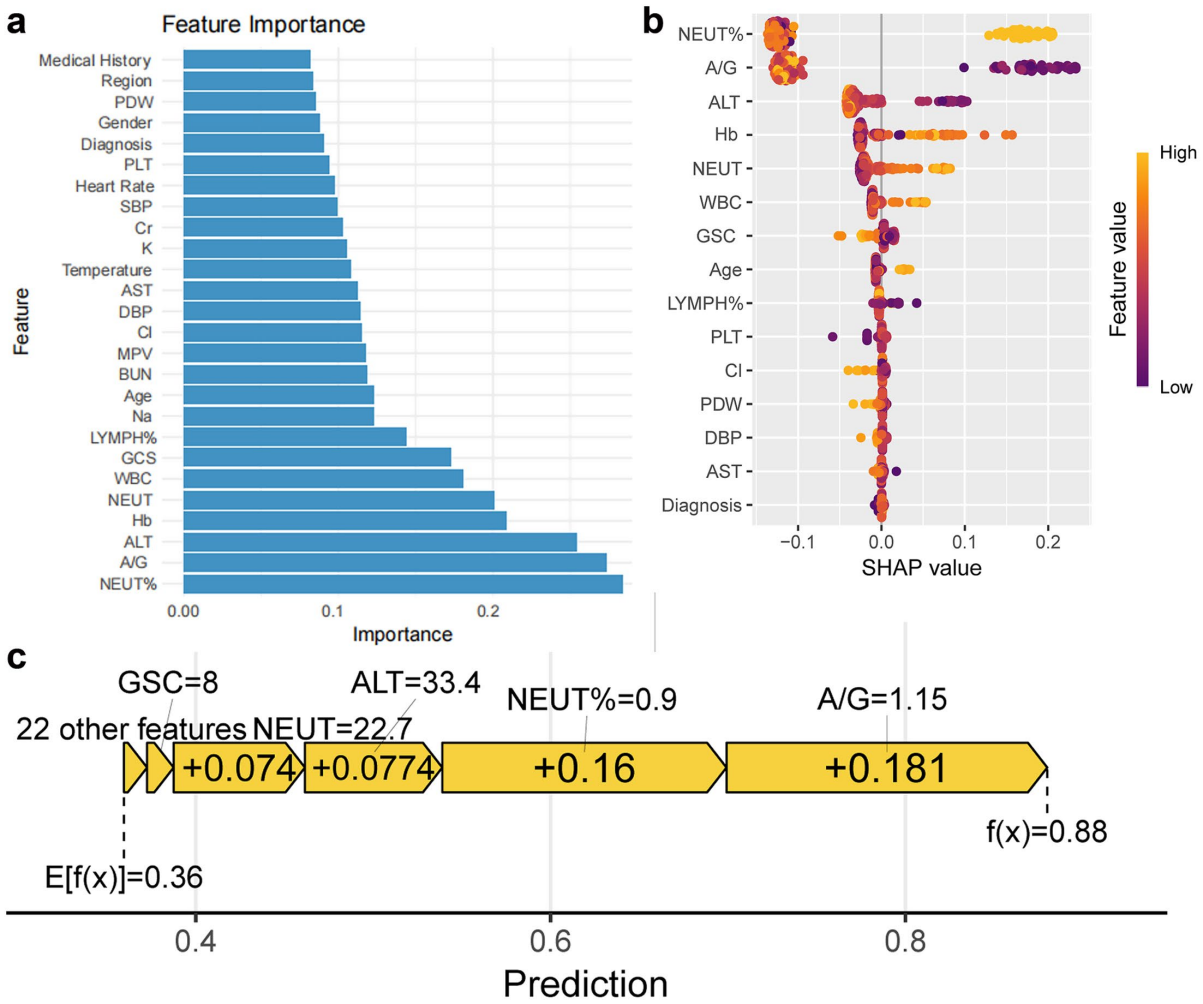
Feature importance of Random Forest model and impact on prediction. **(a)** Bar chart shows feature importance ranking based on optimal parameters of Random Forest model. **(b,c)** Bee swarm plot and force plot show feature impact on predictions.

Random Forest model feature importance analysis revealed neutrophil percentage, A/G ratio, ALT, hemoglobin (Hb), neutrophil count, white blood cell count, GCS score, and lymphocyte percentage as key predictors (Figure 4a). SHAP analysis further revealed: (1) Positive drivers: neutrophil percentage>90% contributed the highest SHAP value, suggesting intense inflammatory response as a core inducer of coagulopathy; A/G < 1.15 and ALT>33.4 U/L indicated liver dysfunction, associated with decreased coagulation factors. Hb < 109.5 g/L and white blood cell count>20.2 × 10^9^/L correlated with increased coagulopathy risk, reflecting contributions of trauma-related blood loss and inflammatory response. (2) Protective factors: GCS score>8 (non-linear relationship) and lymphocyte percentage>15% (anti-inflammatory state) correlated with reduced coagulopathy risk (Figures 4b,c).

## 4 Discussion

This study developed and validated a Random Forest-based machine learning model for predicting coagulopathy risk in emergency department TBI patients. RF demonstrated excellent discriminative ability (AUC = 0.92, median cross-validation AUC = 0.91) and recall (Recall = 0.95, median cross-validation Recall = 0.93), outperforming other candidate algorithms (such as Gradient Boosting Machine and Support Vector Machine). With a training time of only 2.3 min and false negative rate as low as 6%, the model significantly outperforms traditional coagulation tests (such as INR and APTT), which are limited by long processing times and inconsistent diagnostic thresholds (12–14). By integrating routine clinical and laboratory data from emergency departments, this model can rapidly identify high-risk populations for coagulopathy, supporting early intervention and facilitating precise emergency management. These results demonstrate the potential application of machine learning technology in emergency medicine, particularly in time-sensitive TBI treatment scenarios.

### 4.1 Model performance and clinical significance

The excellent performance of the RF model benefits from its powerful capability to process high-dimensional, heterogeneous data (28). The study found that the model’s negative prediction error rate was as low as 6%, which is particularly important in emergency settings, as missed diagnosis of coagulopathy can lead to serious consequences such as hematoma expansion (5, 7, 8). Compared to existing literature, this study’s AUC (0.92) is higher than previous TBI coagulopathy prediction models based on logistic regression (AUC = 0.80–0.85), demonstrating the advantages of ensemble learning methods (6, 17). Additionally, the RF model’s training time was only 15% of the Gradient Boosting Machine’s, which is crucial for emergency prediction systems requiring rapid deployment (29). The model’s robustness was validated through out-of-bag error (OOB error<0.11) and McNemar test (*p* = 0.12), indicating consistent performance across different data subsets and suitability for dynamic emergency environments (30). Furthermore, traditional coagulation tests (CCAs) face issues of inconsistent diagnostic standards and variable positivity rates in TBI patients, whereas this study’s model achieved an AUC of 0.92, significantly outperforming single indicators of CCAs (such as INR or APTT), and integrated dynamic indicators including inflammation and liver function, better aligning with the multi-factorial pathological mechanisms of TBI coagulopathy (12–15). The importance of early identification of coagulopathy in reducing rebleeding risk is well-established; this model’s rapid prediction capability (training time of only 2.3 min) can support emergency departments in initiating targeted treatments (such as tranexamic acid or PCC infusion) within the golden hour, consistent with guideline-recommended “damage control” strategies (31, 32).

Notably, this study prioritized optimizing AUC rather than recall, although the latter is typically more critical in emergency settings (33, 34). This approach was justified as all candidate models maintained high recall levels (0.93–0.96), making AUC optimization more suitable for achieving risk stratification, thereby helping clinicians precisely identify high-risk patients and rationally allocate medical resources. Future research could further explore model performance under different recall thresholds to meet specific clinical needs (such as maximizing sensitivity to reduce missed diagnosis rates).

Additionally, the coagulopathy incidence reported in this study (15.8%) is lower than the 35% mentioned in the introduction (4–6), possibly due to the exclusion of anticoagulant users and adoption of stricter coagulopathy definitions (INR ≥ 1.2 or APTT>34 s). Furthermore, coagulopathy risk increases significantly with trauma severity, and the relatively low proportion of severe TBI in this study sample (approximately 20% of patients with GCS ≤ 8) may further explain the lower overall incidence.

### 4.2 Key predictors and their mechanisms

Feature importance analysis indicates that the percentage of neutrophils, A/G ratio, ALT, hemoglobin (Hb), neutrophil count, white blood cell count, GCS score, and lymphocyte percentage are core factors in predicting TBI-related coagulopathy. Neutrophil percentage was the primary predictor, confirming that the inflammatory cascade reaction triggered by TBI is a core driver of coagulopathy (5, 35, 36). Post-TBI inflammatory response leads to coagulation factor consumption through activation of tissue factor (TF) and endogenous anticoagulation pathways (such as protein C pathway), consistent with the high contribution of neutrophil percentage in this study (37, 38). Additionally, neutrophil extracellular traps (NETs) released by neutrophils may exacerbate hyperfibrinolysis, warranting further investigation (5). Low A/G ratio and high ALT suggest liver dysfunction, which may directly relate to decreased ability to synthesize coagulation factors after TBI (39, 40). This might be associated with the liver’s role in synthesizing anticoagulant factors (such as antithrombin III) (41, 42), consistent with Tsai et al.’s findings that the De Ritis ratio (AST/ALT) correlates with trauma severity, indicating that liver function metrics may serve as biomarkers for coagulopathy risk in TBI patients (43). Low Hb levels, a significant predictive factor, may reflect trauma-associated occult bleeding or inflammation-induced suppression of erythropoiesis (13). Prisco et al. also identified low Hb as a vital predictor of mortality in severe TBI patients, highlighting the strong link between trauma severity and coagulopathy (44). Additionally, patients in the coagulopathy group were older, had lower Hb levels, and poorer GCS scores. These findings align with Depreitere et al.’s research on the vulnerability of elderly trauma patients, suggesting that reduced physiological reserve in older TBI patients may lead to more severe clinical outcomes (45). However, the incidence of coagulopathy among older patients in this study was not significantly increased, likely due to the low proportion of severe TBI cases (approximately 20% with GCS ≤ 8) and the stringent definition of coagulopathy (INR ≥ 1.2 or APTT > 34 s). Furthermore, GCS score>8 and high lymphocyte percentage as protective factors, associated with milder trauma and anti-inflammatory states, may reduce coagulopathy risk by alleviating systemic inflammatory burden (46–48). Severe TBI patients with GCS ≤ 8 have a coagulopathy incidence as high as 60% (49), consistent with this study’s findings (risk sharply increases when GCS ≤ 8). The elevation in white blood cell count further supports the critical role of inflammation in the occurrence of coagulopathy. These indicators are readily obtainable in routine emergency assessments, enhancing the model’s clinical applicability. The predictive factors identified in this study are consistent with existing literature, providing new insights into the pathophysiological mechanisms underlying TBI-related coagulopathy.

### 4.3 Clinical application scenarios

This model can be integrated into emergency decision support systems, supporting the following scenarios: (1) High-risk patient stratification: SHAP value>0.7 corresponds to model prediction probability>0.6; based on cross-validation recall>0.93 and false negative rate<6%, it is suitable for identifying high-risk patients and prioritizing thromboelastography testing to guide individualized blood transfusion (such as fibrinogen concentrate). (2) Dynamic monitoring: inputting updated laboratory data every 6 h within 24 h after admission to dynamically update prediction probabilities and capture delayed coagulopathy. (3) Resource optimization: prioritizing intensive care resources for high-risk patients and optimizing emergency processes. Combined with “damage control” strategies (such as tranexamic acid), effects can be further enhanced (50).

### 4.4 Strengths and limitations

The main strengths of this study include: (1) Utilization of real-world data, enhancing the model’s generalizability; (2) Application of SMOTE technique to address data imbalance, ensuring the model’s predictive ability for the minority class (coagulopathy group) (51, 52); (3) Provision of global and local explanations through SHAP and DALEX analyses, enhancing model interpretability and helping clinicians understand prediction results (53). However, the study also has limitations, including: (1) The data is sourced from a single institution with a limited sample size (n = 322), and there is a significant imbalance between the bleeding disorder group (n = 51) and the non-bleeding disorder group (n = 271), reflecting the actual incidence of TBI-related bleeding disorders (15.8%). Although we addressed the data imbalance using SMOTE to enhance the model’s predictive capability for the minority class (bleeding disorder group), this imbalance may still impact the model’s generalizability, especially in different populations or healthcare settings. Therefore, future multicenter studies should include larger sample sizes and more balanced groups to further validate the model’s robustness and applicability.(2) Exclusion of antiplatelet medications due to missing data, potentially underestimating bleeding risk in some patients. (3) Real-time deployment of the model still requires validation, with future needs for developing user-friendly interfaces and conducting prospective testing to evaluate its performance in actual emergency environments.

### 4.5 Future directions

The RF model in this study provides an effective tool for rapid prediction of TBI coagulopathy, but there remains room for improvement. First, multi-center studies could validate the model’s generalizability and include broader patient populations (such as different races, age groups, or patients with comorbidities). Second, exploration of deep learning methods (such as convolutional neural networks) or time series analysis could capture dynamic changes in coagulation status after TBI. Additionally, combining bedside coagulation testing technologies (such as thromboelastography) might further enhance the model’s real-time capability and accuracy. Finally, developing a clinical decision support system (CDSS) based on this model and evaluating its actual impact on patient outcomes through randomized controlled trials would be an important direction for future research.

## 5 Conclusion

This study demonstrates that a Random Forest-based machine learning model can efficiently predict coagulopathy risk in emergency department TBI patients, with high discriminative ability and clinical utility. Neutrophil percentage, A/G ratio, and ALT play key roles in prediction, suggesting inflammation and liver dysfunction as important drivers of coagulopathy. Despite certain limitations, this model provides a new tool for precise risk stratification in emergency medicine, laying the foundation for improving TBI patient outcomes.

## Data Availability

The original contributions presented in the study are included in the article/Supplementary material, further inquiries can be directed to the corresponding authors.
